# The Quality of Life of a Multidiagnosis Group of Special Needs Children: Associations and Costs

**DOI:** 10.1155/2010/940101

**Published:** 2010-04-18

**Authors:** Sandy Thurston, Louise Paul, Patricia Loney, Maria Wong, Gina Browne

**Affiliations:** ^1^Children's Treatment Network, Simcoe/York, ON, Canada L4M 2Y1; ^2^System-Linked Research Unit on Health and Social Service Utilization, Faculty of Health Sciences, McMaster University, Hamilton, ON, Canada L8P 0A1

## Abstract

*Purpose*. To determine the quality of life, associations, and costs of a multidiagnosis group of special needs children. *Methods*. In this cross-sectional survey families were identified from the Children's Treatment Network, a Canadian multisector program for children with special needs. Families were eligible if the child was aged 2–19 years, resided in Simcoe/York, and if there were multiple child/family needs. Quality of life was measured using the PedsQL (*n* = 429). *Results*. Quality of life scores were lower in this group compared to published healthy and single disorder groups of children. Quality of life scores decreased with advancing age. Child psychosocial well-being was more strongly associated with child/family variables compared to physical well-being. Health Utilization costs were higher in children with greater physical challenges. *Conclusions*. Further research is needed in other complex needs child samples to confirm the decrease in quality of life found in these children into adolescence. Investigations into the interactions of child and family variables are needed.

## 1. Introduction

Recently, the importance of quality of life research has been accepted. Regardless of the disease process, improving a person's quality of life has become an important goal of treatment programs. In the pediatric literature, quality of life studies have been largely confined to single disease states. Usually absent from such studies is an understanding of the quality of life of children with heterogeneous diagnoses often participating in treatment programs with unpredictable prognoses including a deterioration in physical and cognitive function. Varni et al. [1] have recently reported detailed findings on the quality of life (physical and psychosocial) of children and youth both healthy and with varying chronic conditions such as asthma, cancer, cerebral palsy, and psychiatric disorders using the Pediatric Quality of Life Inventory (PedsQL). The quality of life of a complex multidiagnosis group of special needs children and youth has not been reported.

Research is emerging that investigates associations between child/family variables and physical and psychosocial quality of life in special needs children in an attempt to gain a better understanding of the factors influencing child well-being. It is becoming clear that factors affecting a child's physical quality of life differ from those affecting psychosocial quality of life. Studies are also restricted to single disease states making generalizations difficult. In 95 children aged 6–12 with cerebral palsy (CP), Majnemar [2] showed that the strongest predictor of physical functioning (using the PedsQL) was the GMFM (gross motor function measure, *r* = 0.79). The child's general competency in performing tasks (motivation) also correlated with better physical functioning (*r* = 0.45). Psychosocial well-being was associated strongly with few behavior difficulties (*r* = −0.62) and low parent distress (*r* = −0.43). In another CP group (*n* = 39, aged 6–18) autonomous/allowing parenting style was associated with physical (*r* = 0.40) and psychosocial (*r* = 0.40) well-being measured using the Child Health Questionnaire [3]. Arnaud et al. [4] used the Kidscreen quality of life measure in children with CP aged 8–12 (*n* = 818). They showed that children scoring in the lowest quartile for physical function had low gross motor functioning, high pain levels, and high parent stress. Kids in the lowest psychosocial quartile had high pain levels and high parent distress. In children with Attention Deficit Hyperactivity Disorder (ADHD) psychosocial well-being (Child Health Questionnaire) was associated with fewer symptoms of ADHD and less comorbid psychiatric diagnosis [5]. No association was found between reported symptoms of ADHD and co-morbid psychiatric diagnosis and the physical quality of life of these children [5]. An understanding of the associations of child and family variables with child's quality of life (physical and psychosocial) in multidiagnosis special needs population would provide a clearer picture of the issues faced by these individuals and their families. 

Little is known about the relationship between health care expenditures and quality of life of children with special needs. Seid et al. [6] measured the direct costs (excluding pharmacy and mental health) of an inclusive (healthy and special needs) group of children (ages 2–18) that were members of federally supported managed health care plan in San Diego. An inverse relationship between health-related quality of life and direct pediatric health care costs was reported. Seventy percent of this sample had no reported chronic health care conditions. Multiple regression analysis showed that the presence of a chronic health condition and lower physical functioning scale scores consistently accounted for the greatest amount of variance (21.2%) in predicting healthcare costs at 24 months. No research study could be found that related quality of life to direct and indirect health care costs in a group of special needs children from a societal perspective.

The objectives of this paper were threefold: first, to report the quality of life (physical and psychosocial) in a multi-diagnosis, 2–19-year-old group of special needs children/youth; next, to investigate associations between family/child variables with the child's physical and psychosocial quality of life; it was hypothesized that psychosocial quality of life would have stronger associations with child/family variables (parent distress, child behaviors, parenting styles, social support, family functioning, and overall impact on family) than physical quality of life; and finally, to explore trends in the direct and indirect costs associated with quality of life in these families.

## 2. Methods

### 2.1. Research Setting

This descriptive study is part of a cohort study examining the effects and expense of more and less integration of services that provide treatment and rehabilitation for children with complex needs. The cohort is enrolled in the newly modeled Children's Treatment Network (CTN) of Simcoe/York counties in Ontario. The CTN approach is unique in that it is based on the collaboration of numerous existing autonomous, local service agencies utilizing the service coordination, and electronic record functions of the CTN. Ethics approval was obtained for the study by the Research Ethics Board of McMaster University.

### 2.2. Study Design and Procedures

This was a cross-sectional survey of families with a special needs child enrolled in the CTN from May to December 2007. Families were deemed eligible if the child was age 2–19 years, they were residents of Simcoe/York, and there were multiple needs within the family (child's complex needs and/or families needs for example, a parent's medical or mental health problem). The consenting parent/guardian most knowledgeable (PMK) returned a signed consent form to McMaster University indicating their willingness to participate. The PMK then completed a telephone interview (1 hour) by one of three trained interviewers from McMaster University. The size of this convenience sample of PMK completing the interview was 429. 

### 2.3. Measures at 3 Levels

#### 2.3.1. Child

**Child Quality of Life**

The PedsQL is a generic measurement system developed by Varni et al. [7] for use in children ages 2–18 years. The shortened version consists of 15-items comprising three core scales and addresses the physical (5 items), emotional (4 items), social (3 items), and school functioning (3 items) [8]. Item wording differs for children ages 2–4, 5–7, and 8–18. Each item asks how much of a problem it has been during the past month on a five-point scale (0-“never a problem” to 4-“almost always a problem”). The questionnaire appropriate for 8–18 years old was also used for 19 year old children in this study. Parent proxy report formats were used for all eligible children due to the inclusion of children with limited cognitive or communicative abilities. Items are reverse-scored and linearly transformed to a 0–100 scale so that higher scores indicate better quality of life. Psychosocial Quality of Life (10 items) is computed as the sum of the Emotional, Social and School scale scores (range 0–100). Reliability and validity of the shortened version has been documented [8]. The minimally clinically important difference has been reported for the parent proxy total score (Standard Error of the Mean (SEM) = 4.50), psychosocial health score (SEM = 5.49) and physical health score (SEM = 6.92) in a population health survey in the state of California [9].

**Child Behavior**

Behavior is measured using theCanadianNational Longitudinal Survey of Children and Youth (NLSCY) Behavior questionnaire for children ages 2–19 [10]. The questionnaire asks about how the child seems to feel or act regarding age specific behaviors such as getting into fights, inability to sit still, and worrying. The parent is asked to rate the specific behavior from 1-“never” to 3-“often”. Behavior subscales include hyperactivity/inattentive, prosocial, anxiety/emotional disorder, conduct disorder/ physical aggression, indirect aggression, and property offence. Items differ for age groups 2–5 years and 6–19 years. Internal consistency is reported by subscale and age (Cronbachs alpha 0.68–0.84) [11].

**Demographics of the Child**

It includes child age, grade, and PMK report of the main medical and other important diagnosis.

#### 2.3.2. Parent/Guardian Most Knowledgeable

**Health of PMK**

TheKessler scale (K10) [12] measures PMK symptoms of depression and anxiety, a frequent accompaniment of depression. Ten questions measure the frequency of feeling: sad, nervous, restless, hopeless, worthless, everything was an effort, tired for no good reason, so nervous that nothing could calm down, fidgety, so restless could not sit still, or depressed during the past month. Chronic aspects of distress in the past month are examined on a five-point scale (1-“all of the time” to 5-“none of the time”). Reliability and validity have been documented [13]. Scores range for 10–50 where ≤19 indicates no clinically important level of distress, 20–24 indicates mild distress, 25–29 moderate distress, and 30–50 severe distress.

**Parent Wellbeing**

Parents were asked to rate their mental, physical health, and life satisfaction on a five-point scale (1-“very satisfied” or “excellent” to 5-“very dissatisfied” or “poor”). These questions were taken from the Canadian Community Health Survey (CCHS 2.2) [14].

**Parenting Practices**

The NLSCY Parenting Scale was used and consists of twenty-five questions adapted from the Parent Practices Scale [15]. The following four parenting behaviors were measured: positive interaction (praise, play), hostility (anger, discipline), consistency (follow through), and punitive (yelling, physical punishment). PMK rated each item (e.g., “Do something special with your child that he/she enjoys”) in terms of frequency from 0-“never” to 4-“many times each day”. Higher scores indicate greater frequencies for each type of parenting behavior. Internal consistency is reported by subscale and age group (Cronbachs alpha 0.39–0.75) [11].

#### 2.3.3. Family

**Family Functioning**

Thirteen items taken from the NLSCY population survey [10], based on a subscale of the McMaster Family Assessment Device [16], were used to gather information on various aspects of family functioning, namely, problem solving, communication, roles, affective responsiveness, affective involvement, and behavior control. PMK rated each item (e.g., “We avoid discussing our fears or concerns”) along a four-point scale from 0-“strongly agree” to 3-“strongly disagree”. Negatively oriented items are reverse scored so that higher scores represent greater family dysfunction. The measure has internal consistency (chronbach's alpha = 0.86) [16]. Scores range from 0 to 36 with scores ≥15 indicating family dysfunction.

**Social Support**

The level of social support of the PMK was assessed using an eight item shortened version of the Social Provisions Scale [17]. Different social support constructs were measured: guidance, reliable alliance (i.e., feeling assured that others would be available to offer practical help), and attachment. PMK rated each item along a four-point scale from 0-“strongly disagree” to 3-“strongly agree”. Higher scores represent greater social support. The reliability and validity have been reported [17]. The total score ranges from 0 to 24.

**Caregiver Burden**

The Impact on Family (IOF) Scale determines the effects of a chronic illness on parents and families. Parents respond on a four-point scale to the degree that statements apply to their family (1-“strongly agree” to 4-“strongly disagree”) [18]. The revised IOF scale (15 items) has been validated [19, 20]. Statements cover four dimensions: financial burden, family/social impact, personal strain, and mastery (e.g., fatigue is a problem, see family and friends less, need to change plans at last minute, little desire to go out).

**Demographics of the Family**

A standard form including spiritual or faith orientation, ethnicity, and languages was selected from the Canadian National Longitudinal Survey on Children and Youth (NLSCY) that also includes community dwelling disabled children [10]. Sociodemographic data were gathered on the PMK gender, age, and educational level as well as on household income and family status.

**Costs for Health and Social Services**

Health and social service utilization is measured by an inventory developed initially by Browne et al. [21] and is currently updated to be the Expenditures for Health and Social Service Utilization Questionnaire [22]. This measure has consistently distinguished expenditures for use of services by youth with and without behavior problems, people with and without mental illness, with and without a range of chronic diseases, with and without poverty [23, 24]. This tool was developed as a modification of Spitzer's work [25]. It consists of questions about the respondent's use of eight categories of direct health services: primary care, emergency room, specialists, hospital episodes, hospital days (irrespective of episode), emergency room specialists, seven types of other community health professionals, and laboratory services. Recall that data are used in order to assess the patient's use of all health services. Inquiries are “restricted to the reliable duration of recall span: 6-months for remembering a hospitalization, 2 weeks for a visit to a physician, and 2 days for the consumption of a prescription medication”. To calculate 6-month utilization, the various spans of time are extended to yield a 6-month rate of utilization per category of health service, as proposed by Spitzer [25] and Petrou et al. [26]. The 6 month rate per category of service is multiplied by the 2006 unit cost (Canadian $) for that service. This approach to the measurement of costs was recently acknowledged by Guerriere et al. [27, 28] as one of the few measures of ambulatory utilization published and empirically validated.

## 3. Analysis

Descriptive statistics (numbers, percentages, means, and standard deviations) were calculated for demographic data, child/family variables, and expenditures. The behavior subscale measures have different numbers of items applicable to different age groups: children 2 to 5 years, and children and youth 6 to 19 years. This resulted in a changing number of participants for the behavior variables. The behavior scales for different age groups were transformed using the interpolation technique where the mean of the behavior scale scores for children 2 to 5 years with fewer items was multiplied times the number of items for older children. This transformed mean was used in the analysis. In 18 instances, there were reports on two or three children with complex needs in the same family and only one report of parent variables. In these instances, the PMK was counted 2 or 3 times as appropriate to ensure a matched number of children and parents in the analysis. 

Pearson correlation coefficients were calculated for quality of life variables and other child/family variables. Differences between dichotomized Psychosocial (≥3 or <3 often or almost always responses) and Physical (≥2 or <2 often or almost always responses) quality of life and family/child variables were compared using Chi-square and *t*-tests. These cut-offs were chosen as it was thought to represent a child with clinically important psychosocial issues, requiring professional follow-up and clinically important physical restrictions. Expenditure variables were skewed; so the nonparametric Kruskal-Wallis test was used. 

## 4. Results

Table 1 shows the demographic characteristics of participating families. The majority of PMK were mothers of the children (85%), born in Canada (75%), and spoke English (90%). The average PMK was 41 years, 90% were female, 85% were married/common-law, 69% were employed, and the median household income was $60–$69,000. There was an even split between families residing in Simcoe (52%) and York (49%) county. The average child age at interview was 8 years with 66% of the sample being male. Thirty-eight percent of the children were in preschool (up to and including Kindergarten), 36% in grade 1–5 (elementary), and 26% in grade 6 and up (junior). Sixty percent of children were receiving service from Community Care Access Centres and School Boards at time of entry into the CTN. The top PMK reported diagnoses for the children were mental and behavioral disorders (85%), one of which was autism (25%); diseases of the nervous system (34%); and congenital malformations, deformations, and chromosomal abnormalities (19%) (Table 2). Fifty one percent of children had more than one reported medical problem.

Child quality of life scores is presented in Table 3 (*n* = 429). There was a significant trend of decreasing total quality of life by increasing age. These differences exceeded minimally clinically important differences (MCIDs) reported by Varni et al. [9] between the three age groups. Physical well-being was the lowest in the oldest age group (grades 6&up) and psychosocial well-being was the lowest in school aged children (grades 1–5 and grades 6&up). These differences also exceeded the MCID. 

Within this population of children with complex needs there was no correlation between the child's physical functioning and their psychosocial functioning (Table 4). Physical well-being showed moderate associations with less adverse impact on the family and the presence of prosocial behavior (*r* = 0.3–0.5) [29]. The child's physical functioning had weak associations (*r* = 0.1 − <0.3) with social support, parental distress and life satisfaction, hostile and consistency parenting styles, family functioning, and negative child behaviors. Psychosocial well-being in the children/youth was strongly associated (*r* > 0.5) with reports of fewer anxiety/emotional behaviors. Psychosocial well-being was moderately associated with lower levels of adverse family impact, less hostile parenting, less child hyperactivity, conduct disorder, indirect aggression, and property offence behaviors. Parental distress and life satisfaction, positive parenting styles, and family functioning showed weak associations with child psychosocial functioning (*r* = 0.1 − <0.3). In this sample, the child's psychosocial functioning was unrelated to PMK punitive (emotionally charged) parenting or prosocial behavior. However, 67% of punitive parenting levels were none to mild, 33% moderate, and 0% severe in this sample.

Table 5 presents family/child variables dichotomized by responding often or almost always to ≥2 or <2 items (5 total) in the physical function domains. In children with ≥2 or more identified physical challenges there were lower levels of PMK social support, greater overall adverse impact on the family, less consistent parenting, less hostile parenting, and less prosocial and hyperactivity behaviors in the children. These differences were all statistically significant. The number of physical challenges in the children was not related to family function, child anxiety, conduct disorder, property offense, parent symptoms of depression/anxiety, positive, nor punitive parenting.

Table 6 presents family/child variables dichotomized by responding often or almost always to ≥3 or <3 items (10 total) about the child's emotional, social, and school function domains. In children with ≥3 or more identified psychosocial issues there was less PMK social support, greater report of adverse impact on the family, and poorer overall family functioning. Parent distress was higher, parenting practices (positive interaction, consistency, hostile) were worse, and child behavior scores were all higher (poorer) in the children and youth with poor psychosocial function. These differences were all statistically significant. Prosocial behavior was also higher in children and youth with fewer symptoms of psychosocial problems. 

Table 7 shows per 6 month expenditures for the child's use of human services by their physical function. Although overall primary care use was not statistically significant, in the group with ≥2 challenges there was greater use of ambulance and 911 calls. Overall physician specialist costs were higher in this group. Economically important higher use of Neurologists and Pediatricians was observed. Economically and statistically significant higher use of physiotherapists, nutritionists, nurses, Personal Support Workers and Special Ed. Services was seen. The overall costs of other health and social support services were not different between the groups largely due to the greater increased use of social and recreations programs in children less physically challenged (<2 items) group. Community supports, outpatient lab test costs, medication and supply costs, and hospital and respite costs were all higher in the group with ≥2 physical challenges. These differences were all statistically significant.

Table 8 shows per 6 month expenditures for the child's use of human services by their psychosocial well-being. Total primary and secondary care costs were similar between children exhibiting ≥3 or <3 psychosocial behaviors. Total other health and other service provider costs were similar between the two groups; however, economically important increased use of psychologists, chiropractors, and mental health counselors was noted in the group exhibiting more psychosocial distress. Also, greater use of physiotherapists, nutritionists, nurses, personal support workers, daycare, and device costs was seen in the group of children with less psychosocial distress. 

## 5. Discussion

This is the first study to present quality of life scores in a heterogeneous group of special needs children ages 2–19. Quality of life scores were much lower than reported mean scores for healthy children (physical score 87.84, psychosocial 81.87) [1]. They were also lower than scores reported by 10 disease clusters (physical score range 64.40–85.89, psychosocial range 67.46–77.34) [1]. The scores obtained in this multidiagnosis sample were comparable to reported scores for children with Cerebral Palsy attending a CP clinic in San Diego [30] (physical 43.19, psychosocial 55.91). Children in Varni's CP sample [30], however, were excluded if they were not able to self-report PedsQL scores. Generally the physical score in the CP group is lower likely because of the inclusion of children with quadriplegia while the psychosocial score is higher likely due to our inclusion of kids with mental and behavioral diagnosis. The lower scores illustrate the multifaceted needs and issues faced by this heterogeneous group of children and youth with complex needs usually excluded from other studies. 

The finding of declining physical and psychosocial well-being in these children/youth with advancing age is troubling. Perhaps this finding demonstrates the value of the recent early intervention services in Ontario aged 0–6 years and captures the population of adolescents that did not receive this. Perhaps, fewer formal services/programs are available for older youth thus possibly explaining their declining physical function or perhaps older children simply decide not to pursue further efforts that maintain/improve the physical aspects of their lives. Nevertheless, the CTN decision to include children up to age 19 is supported by this finding. The decline in psychosocial function could be the results of accumulating parent distress, less favorable parenting styles, and/or the child exclusion from peers and activities when in school. 

The psychosocial well-being of complex needs children was more strongly related to child and family variables than physical functioning in this sample. Lower psychosocial functioning was associated with more negative child behaviors and hostile parenting behavior. There was a weak association with punitive parenting, however, which is likely explained by the absence of severe punition in this sample. These findings are similar to previous reports in special needs populations of parent distress, negative child behavior, and negative parenting practicing being associated with lower child psychosocial quality of life [2–5]. There may be complex interactions between physical and psychosocial factors within the child and parent that could lead to parent distress, ineffective parenting, child behavior, and family functioning. It is the associations and interactions of these variables overtime that help explain the child's quality of life. This analysis is the purpose of our next paper.

The increased use of specialist costs in children with greater physical need is not surprising nor is the increased use of lab tests, medications, community support, special ed., and hospital and respite stays. The overall costs to the health care system in children with greater physical disability, however, are largely offset by the increased use of social and recreation programs by those children less affected. The more physically affected children are not using the social and recreational sector to the same extent than those less affected. The presence of psychosocial problems was not associated with a greater use of any services. Economic trends of increased use of mental health professionals, however, are seen in children with more psychosocial problems. The use of special education services was similar between the two psychosocial groups perhaps indicating under recognition of psychosocial issues in school aged kids. This is the first comprehensive presentation of health service utilization data in relation to the quality of life of children with complex needs.

Results and findings are difficult to generalize outside of this study population as contexts may differ. The PMK in this sample were predominantly married, educated, working mothers. This study may be missing important information from working, lower educated, single parents and their children, likely those with greater need and harder to reach. Also, quality of life data were parent-reported. Generally, parents underestimate their child's quality of life compared to child self reports [1]. Therefore, associations may differ when child self report data are used, particularly for older kids. It was not feasible to obtain self-report data from this complex needs group due to the wide range of limitations present and budget constraints of the study.

## 6. Conclusions

Quality of life scores decreased with advancing age in these complex, multidiagnosis children with special needs aged 2–19 years. Psychosocial quality of life showed stronger univariate associations with child and family variables measured compared with physical quality of life. Health and Social Service Utilization costs were higher in children with greater physical challenges mainly due to increased use of medications, treatment, supplies, and aids. 

## Figures and Tables

**Table 1:** Characteristics of sample (*n* = 429).

Variable		
*Respondent (PMK)*		
Age	Years, mean (SD)	40.72 (7.60)
Gender	Female, *n* (%)	386 (90.0)
Relationship to child	Mother, *n* (%)	366 (85.3)
Marital Status	Married, *n* (%)	365 (85.1)
Employment status	Employed, *n* (%)	396 (69.0)
Country of Birth	Canada, *n* (%)	323 (75.3)
Household language	English, *n* (%)	387 (90.2)
Household income	median	$60–69,000
PMK Level of Education	median	Completed postsecondary
PMK location of home	Simcoe, *n* (%)	221 (51.5)

*Child *		
Age	Years, mean (SD)	8.18 (4.36)
Gender	Male, *n* (%)	285 (66.4)
Status	Preschool, *n* (%)	164 (38.2)
	Elementary, *n* (%)	154 (35.9)
	Junior, *n* (%)	111 (25.9)
Grade	median	grade 2
Service Provider	Early Intervention, *n* (%)	143 (33.4)
	CCAC & School, *n* (%)	259 (60.4)
	New CTN referral, *n* (%)	27 (6.3)

**Table 2:** PMK reported child diagnosis (*n* = 429).

ICD-10 Diagnostic category		Count	%
A00-B99	Infectious and parasitic diseases	3	0.7
C00-D48	Neoplasm	4	0.9
D50-D89	Diseases of the blood & blood forming organs involving immune mechanism	7	1.6
E00-E90	Endocrine, nutritional and metabolic diseases	17	4.0
F00-F99	Mental and behavioral disorders	363	84.6
	Autism	109	25.4
	Unspecified Disorder of psychological development	77	18.0
	Specific developmental disorders of Speech and Language	46	10.7
	Hyperkinetic disorders (ADD/ADHD)	44	10.3
G00-G99	Disease of Nervous system	144	33.6
	Cerebral Palsy	71	16.6
	Epilepsy	37	8.8
H00-H59	Disease of eye and adnexa	20	4.7
H60-H95	Disease of the ear and mastoid process	10	2.3
I00-I99	Disease of circulatory system	10	2.3
J00-J99	Diseases of respiratory system	25	5.8
K00-K93	Disease of digestive systems	4	0.9
L00-L99	Diseases of the skin and subcutaneous tissues	3	0.7
M00-M99	Diseases of the musculoskeletal system and connective tissues	10	2.3
N00-N99	Diseases of genitourinary system	2	0.5
P00-P99	Certain conditions originating in the perinatal period	7	1.6
Q00-Q99	Congenital malformations, deformations, and chromosomal abnormalities	83	19.4
	Down's syndrome	29	6.8
R00-R99	Symptoms, signs and abnormal clinical and laboratory findings, not elsewhere classified	22	5.1
S00-T99	Injury, poisoning, and certain other consequences of external causes	23	5.4

**Table 3:** Child Quality of Life scores.

PedsQL mean (SD)	Total sample *n* = 429	Preschool *n* = 164	Grade 1–5 *n* = 154	Grade 6& up *n* = 111	*F*	*P*
Total Score	57.86 (16.87)	62.91 (15.70)	57.66 (16.69)	50.68 (16.26)	18.881	<.001
Physical	55.77 (33.87)	59.65 (33.22)	60.71 (31.69)	43.15 (34.83)	10.905	<.001
Psychosocial	59.10 (18.62)	64.85 (16.04)	56.29 (18.80)	54.49 (19.84)	13.768	<.001

**Table 4:** Correlations between Quality of Life scores and child/family variables.

	PedsQL scores			
*Child variables*	Physical	Psychosocial	Total	*n*

Psychosocial Quality of Life	0.010	—	—	429
Pro-social Behavior	0.293**	0.046	0.236**	267
Hyperactivity/Inattention	0.157**	−0.482**	−0.237**	425
Anxiety/Emotional Disorder	−0.023	−0.669**	−0.499**	428
Conduct Disorder & Physical Aggression	0.141**	−0.347**	−0.153**	425
Indirect Aggression	0.076	−0.341**	−0.200**	264
Property Offence	0.103	−0.389**	−0.215**	267

*PMK variables*				
Mental health	−0.130*	−0.199**	−0.229**	428
Physical health	−0.070	−0.079	−0.102*	428
Life satisfaction	−0.108*	−0.230**	−0.237**	428
K10	−0.113*	−0.284**	−0.278**	429
Positive interaction Parenting	−0.084	0.246**	0.119*	429
Hostile/ineffective parenting	0.144**	−0.430**	−0.208**	426
Consistency parenting	0.227**	0.170**	0.272**	418
Punitive (Adverse) parenting	0.012	0.046	0.038	425

*Family variables*				
Family Functioning	−0.072	−0.230**	−0.211**	429
Social Support	0.173**	0.244**	0.293**	429
Impact on Family	0.311**	0.302**	0.433**	429

**Correlation is significant at the 0.01 level (2-tailed).

*Correlation is significant at the 0.05 level (2-tailed).

**Table 5:** Family and child variables by Physical Quality of Life.

Variable (range)	Total Sample		≥2 physical problems		<2 physical problems		*t*	*P*
	Mean (SD)	*n*	Mean (SD)	*n*	Mean (SD)	*n*		
*Social Support; high score indicates the presence of support*								
Social Support (0–24)	17.53 (4.55)	429	16.8 (4.39)	190	18.10 (4.60)	239	−2.977	.003

*Family Functioning; high score indicates family dysfunction*								
Family Functioning (0–36)	9.29 (6.15)	429	9.45 (6.02)	190	9.16 (6.27)	239	0.482	.630

*Impact on Family; high score reflects less negative impact*								
Impact on Family (0–45)	24.14 (9.99)	429	21.38(9.20)	190	26.33 (10.07)	239	−5.306	<.001

*Child Behavior; high score indicates presence of the behavior*								
Hyperactivity (0–16)	7.5 (3.83)	425	6.87 (4.00)	186	7.99 (3.63)	239	−3.024	.003
Prosocial (0–20)	10.33 (5.72)	267	9.27 (5.79)	123	11.24 (5.52)	144	−2.852	.005
Anxiety (0–14)	3.82 (2.97)	428	3.82 (3.08)	189	3.82 (2.88)	239	0.029	.977
Conduct disorder (0–12)	2.29 (2.77)	425	2.01 (2.64)	186	2.52 (2.84)	239	−1.905	.057
Indirect Aggression (0–10)	0.96 (1.64)	264	0.88 (1.76)	120	1.03 (1.53)	144	−0.723	.470
Property Offence (0–12)	1.57 (2.01)	267	1.38 (1.95)	123	1.72 (2.05)	144	−1.382	.168

*Parent distress; high score indicates more distress*								
K10 (10–50)	20.01 (6.55)	429	20.46 (7.32)	190	19.66 (5.86)	239	0.216	.81

*Parenting Style; high score indicates presence of style*								
Positive (0–20)	15.07 (3.01)	429	15.34 (3.06)	190	14.86 (2.96)	239	1.659	.098
Consistent (0–20)	13.46 (3.86)	418	12.76 (4.13)	179	13.99 (3.56)	239	−3.194	.002
Hostile (0–28)	10.33 (4.79)	426	9.66 (4.98)	187	10.86 (4.57)	239	−2.571	.010
Punitive (0–20)	9.52 (2.05)	425	9.51 (2.04)	186	9.52 (2.04)	239	−0.029	.977

*Parent well-being; high score indicates lack of well-being*								
Life satisfaction (0–5)	1.92 (0.871)	428	1.96 (0.978)	190	1.89 (0.791)	238	0.798	.426
Physical health (0–5)	2.48 (1.061)	428	2.55 (1.086)	190	2.46 (1.034)	238	0.828	.408
Mental health (0–5)	2.30 (1.016)	428	2.44 (1.031)	190	2.22 (0.999)	238	2.268	.024

**Table 6:** Family and child variables by Psychosocial Quality of Life.

Variable (range)	Total		≥3 psychosocial problems		<3 psychosocial problems		*t*	*P*
	Mean (SD)	*n*	Mean (SD)	*n*	Mean (SD)	*n*		
*Social Support; high score indicates the presence of support*								
Social Support (0–24)	17.53 (4.55)	429	16.22 (4.69)	175	18.43 (4.23)	254	−5.090	<.001

*Family Functioning; high score indicates family dysfunction*								
Family Functioning (0–36)	9.29 (6.15)	429	10.86 (6.24)	175	8.20 (5.86)	254	4.502	<.001

*Impact on Family; high score reflects less negative impact*								
Impact on Family (0–45)	24.14 (9.99)	429	20.80 (9.06)	175	25.38 (9.92)	254	−5.959	<.001

*Child Behavior; high score indicates the presence of the behavior*								
Hyperactivity (0–16)	7.5 (3.83)	425	9.63 (3.23)	175	7.00 (3.72)	250	5.457	<.001
Pro-social (0–20)	10.33 (5.72)	267	9.48 (5.40)	134	11.19 (5.92)	133	−2.473	.014
Anxiety (0–14)	3.82 (2.97)	428	5.7 (3.14)	175	2.52 (1.99)	253	11.837	<.001
Conduct disorder (0–12)	2.29 (2.77)	425	3.34 (2.99)	175	1.56 (2.34)	250	6.565	<.001
Indirect Aggression (0–10)	0.96 (1.64)	264	1.32 (1.89)	133	0.60 (1.25)	131	3.695	<.001
Property Offence (0–12)	1.57 (2.01)	267	2.25 (2.38)	134	0.88 (1.21)	133	5.919	<.001

*Parent distress; high score indicates more distress*								
K10 (10–50)	20.01 (6.55)	429	22.03 (7.10)	175	18.63 (5.76)	254	5.257	<.001

*Parenting Style; high score indicates presence of style*								
Positive interaction (0–20)	15.07 (3.01)	429	14.35 (3.26)	175	15.57 (2.72)	254	−4.076	<.001
Consistent (0–20)	13.46 (3.86)	418	12.66 (4.09)	175	14.04 (3.58)	243	−3.569	<.001
Hostile (0–28)	10.33 (4.79)	426	12.43 (4.93)	175	8.87 (4.10)	251	7.858	<.001
Punitive (0–20)	9.52 (2.05)	425	9.43 (1.95)	175	9.58 (2.12)	250	−0.718	.473

*Parent well-being; high score indicates lack of well-being*								
Life satisfaction (0–5)	1.92 (0.871)	428	2.16 (1.03)	174	1.77 (0.72)	254	4.301	<.001
Physical health (0–5)	2.48 (1.061)	428	2.56 (1.10)	174	2.46 (1.02)	254	1.024	.306
Mental health (0–5)	2.30 (1.016)	428	2.48 (1.08)	174	2.21 (0.96)	254	2.697	.007

**Table 7:** Mean 6-month cost of child use of Health and Social Services Utilization by Physical Quality of Life.

	Physical Problems				Test Statistic: Kruskal	
	≥2 (*n* = 190)		<2 (*n* = 239)		Wallis Test	
	Mean	S.D.	Mean	S.D.	*χ* ^2^	*P*
*Direct Costs*						

*Primary Care Provider visits*						
(a) Family Physician/Walk in Clinic (Primary care)	75.68	118.84	60.84	89.28	0.303	.582
(b) Emergency Room visits	74.44	169.79	53.54	134.48	1.952	.162
(c) 911 calls	0.74	4.09	0.1	1.51	4.938	.026
(d) Ambulance Service	10.11	48.33	1	15.52	7.394	.007

*Primary Care Provider Services*	160.97	237.55	115.48	181.48	2.894	.089

*Physician Specialist visits*						
(e) Adolescent Medicine Allergist	1.46	13.34	0.47	5.08	0.511	.475
(f) Cardiologist	4.8	18.65	2.8	28.54	5.899	.015
(g) Dermatologist	0.56	4.47	0	0	3.791	.052
(h) Ears/Nose/Throat Specialist	8.58	23.29	9.04	22.18	0.371	.543
(i) Endocrinologist	3.88	20.91	2.25	17.25	0.990	.320
(j) Gastroentologist	3.2	17.39	1.02	11.09	4.117	.042
(l) Infectious Disease/HIV Specialist	0.29	4.04	0	0	1.258	.262
(m) Hematologist or Oncologist	5.12	26.76	1.78	23.89	6.392	.011
(n) Nephrologist	3.18	17.3	0.28	4.34	6.171	.013
(o) Neurosurgeon Orthopedics/Neurologist	36.46	78.52	10.68	47.36	30.259	.000
(p) Ophthalmologist	18.19	38.61	5.67	19.96	20.363	.000
(q) Pediatrician	106.89	400.52	56.27	97.16	5.044	.025
(r) Psychiatrist	17.33	117.93	4.24	38.7	3.756	.053
(s) Respirologist	6.4	44.17	2.8	19.49	0.967	.326
(t) Rheumatologist	6.72	88.25	1.27	16.19	0.054	.816
(u) Rehabilitation Doctor	0	0	0.26	4.03	0.795	.373
(v) Surgeon (general, dental)	1.16	8.42	1.85	12.01	0.290	.590
(w) Surgeon (orthopedic)	10.57	36.89	1.94	13.62	12.056	.001
(x) Surgeon (neurological)	2.24	16.97	0.25	3.93	2.622	.105
(y) Other health professional visit cost	75.57	178.76	34.94	136.26	28.183	.000

*Physician Specialist cost*	312.59	545.41	137.78	204.44	38.598	.000

*Other Health and/or Social services providers*						
(a) Physiotherapist	627.51	1138.9	119.28	530.35	86.461	.000
(b) Massage Therapist	35.74	264.71	3.93	31.96	3.381	.066
(c) Occupational Therapist	558.7	1392	378.31	746.29	1.074	.300
(d) Speech Language Pathologist	435.08	1021.5	505.68	904.37	0.852	.356
(e) Chiropractor	23.77	153.39	9.47	51.66	1.512	.219
(f) Psychologist	44.74	270.9	32.81	144.44	0.000	.993
(g) Podiatrist/Chiropodist	8.95	70.47	3.2	27.36	1.019	.313
(h) Nutritionist/Dietician	48.69	184.22	15.67	81.76	11.371	.001
(i) Nurse Practitioner	13.89	133.92	0.35	4.21	1.266	.260
(j) Visiting Nurses (Home Care/PHN/VON/SEN)	790.81	4803.1	23.56	220.26	9.263	.002
(k) Private Nurse	373.26	4767.4	0	0	2.522	.112
(l) Optometrist	18.5	41.34	31.8	152.99	1.001	.317
(m) Dentist	129.14	160.2	126.89	134.77	0.029	.864
(n) Social Worker	26.84	135.2	31.15	231.6	0.000	.997
(o) Children's Aid Worker	0	0	18.8	221.07	4.012	.045
(p) Adolescence/School Counselor	2.52	27.41	3	46.45	0.603	.437
(q) Family Counselor	5.36	42.56	9.94	119.03	0.006	.939
(r) Mental Health Counselor	16.38	225.74	14.52	188.35	0.596	.440
(s) Homemaker/Personal Support Worker	1312.7	8672.2	197.32	2002.1	16.596	.000
(t) Child/Daycare	471.87	1783.1	660.7	3453.2	2.938	.087
(u) Subsidized Daycare	38.64	343.66	53.73	454.04	0.014	.905
(v) Naturopath/Homeopath	3.37	24.29	10.14	52.35	3.071	.080
(w) Complementary Therapy	27.95	244.5	0.77	8.81	1.249	.264
(aa) Police	0	0	1	12.26	1.594	.207
(dd) Social and Recreation Programs	10939	35075	14468	36389	6.479	.011
(ee) Community Support Programs	5.92	81.62	17.07	144.62	1.882	.170
(ff) Special Education Services	1270.8	1445.4	945.46	1350.8	5.130	.024
(gg) Other Special Education Supports	139.26	693.48	146.91	435.03	3.541	.060
(hh) Others Social and Health providers	970.35	4889.1	960.18	3402.8	0.560	.454

*Other Health and Social providers cost*	18340	39337	18790	37367	0.067	.796

*Community Support Services*						
(a) Groups/Peer Support	0.18	1.66	0.13	1.94	1.522	.217
(c) Transportation Services	22.87	62.9	3.15	22.33	20.245	.000
Other community Support Services	30.99	127.46	37.57	162.39	0.031	.859

*Total Community Support Services*	54.04	137.37	40.84	163.17	8.006	.005

*Outpatient lab tests*						
(a) Blood	27.68	77.89	10.4	36.36	17.090	.000
(b) Specimens	6.37	31.89	2.34	9.47	3.920	.048
(c) Scopes	0	0	0.65	10.04	0.795	.373
(d) X-rays	10.9	30.64	3.78	14.97	11.765	.001
(e) Scans	10.56	37.2	4.2	22.5	4.994	.025
(f) Breathing tests	1.29	6.53	0.6	4.74	2.339	.126
(g) ECG	1.35	6.13	0.17	1.81	7.654	.006
(h) EEG	2.41	11.5	1.67	10.98	0.947	.331
(i) EMG	1.61	15.65	0	0	2.522	.112
(l) Other outpatient tests	18.97	67.87	11.81	45.25	0.955	.329

*Outpatient laboratory tests*	81.15	172.97	35.62	77.76	17.343	.000

*Medications, treatments and Supplies/Aids*						
Medication	628.84	1982	218.17	720.14	21.649	.000
Treatment costs total	157.34	1983	15.05	151.71	1.170	.279
Supply and device cost	1691.5	8221.9	75.76	366.7	78.729	.000

*Medication, treatments, supplies, device, aids*	2477.6	8844	308.98	859.97	63.309	.000

*Direct Costs excluding Hospital stay, Day surgery*						
Direct Costs excluding Hospital	21426	40555	19429	37390	1.712	.191

*Hospital, Day surgery facility*						
Hospital Cost	413.55	1474.3	296.95	2467.8	6.643	.010
Day Surgery Facility Stay cost	20	63.58	10.04	43.77	3.257	.071
Respite	682.27	2170.7	257.97	952.26	7.280	.007

*Direct Costs including Hospital stay, Day surgery*						
Total Direct Costs including Hospital/respite	22542	40525	19994	37448	3.010	.083

**Table 8:** Mean 6-month cost of child use of Health and Social Services by Psychosocial Quality of Life.

	Psychosocial Problems				Test Statistic: Kruskal	
	≥3 (*n* = 175)		<3 (*n* = 254)		Wallis Test	
	Mean	S.D.	Mean	S.D.	*χ* ^2^	*P*
*Direct Costs*						

*Primary Care Provider visits*						
(a) Family Physician/Walk in Clinic (Primary care)	70.22	101.79	65.48	104.91	1.206	.272
(b) Emergency Room visits	52.6	143.8	69.82	156.17	2.235	.135
(c) 911 calls	0.13	1.76	0.55	3.55	2.065	.151
(d) Ambulance Service	1.37	18.14	7.56	42	3.345	.067

*Primary Care Provider Services*	124.32	193.84	143.41	219.11	0.005	.946

*Physician Specialist visits*						
(e) Adolescent Medicine Allergist	0.95	12.62	0.88	6.94	0.887	.346
(f) Cardiologist	2.43	13.6	4.55	29.98	0.433	.51
(g) Dermatologist	0.41	3.81	0.14	2.24	0.835	.361
(h) Ears/Nose/Throat Specialist	9.75	25.94	8.21	20.1	0.119	.73
(i) Endocrinologist	2.3	15.91	3.43	20.8	0.287	.592
(j) Gastroentologist	3.12	18.73	1.2	10.04	0.86	.354
(m) Hematologist or Oncologist	0	0	0.22	3.49	0.689	.407
(n) Nephrologist	2.78	21.42	3.59	27.58	0.091	.763
(o) Neurosurgeon Orthopedics/Neurologist	3.07	17.35	0.53	5.94	3.967	.046
(p) Ophthalmologist	20.83	65.7	22.96	63.43	0.819	.366
(q) Pediatrician	8.27	29.52	13.24	30.74	6.221	.013
(r) Psychiatrist	61.55	105.34	90.49	349.02	0.436	.509
(s) Respirologist	17.73	124.54	4.74	33.67	3.238	.072
(t) Rheumatologist	2.43	18.83	5.74	39.65	2.183	.14
(u) Rehabilitation Doctor	7.64	92.04	0.96	15.25	1.945	.163
(v) Surgeon (general, dental)	0.36	4.71	0	0	1.451	.228
(w) Surgeon (orthopedic)	1.77	10.97	1.39	10.3	0.106	.745
(x) Surgeon (neurological)	4.12	22.38	6.89	29.57	2.112	.146
(y) Other health professional visit cost	0.69	9.19	1.44	13.15	0.895	.344

*Physician Specialist cost*	59.11	171.06	48.68	147.85	0.029	.866

*Other Health and/or Social services providers*						
(a) Physiotherapist	221.09	633.74	431.51	1028.1	9.94	.002
(b) Massage Therapist	29.37	239.68	10.2	118.61	0.993	.319
(c) Occupational Therapist	485.35	1197.5	436.86	997.59	0.029	.865
(d) Speech Language Pathologist	606.55	1313.6	427.56	822.96	0.007	.933
(e) Chiropractor	32.67	165.83	4.19	30.44	7.505	.006
(f) Psychologist	61.81	307.15	21.75	94.68	0.832	.362
(g) Podiatrist/Chiropodist	7.77	45.81	4.35	54.58	3.584	.058
(h) Nutritionist/Dietician	14.47	73.72	41.19	167.53	4.267	.039
(i) Nurse Practitioner	10.02	132.49	3.82	37.44	1.44	.23
(j) Visiting Nurses (Home Care/PHN/VON/SEN)	254.03	2541.4	438.7	3615.2	1.686	.194
(k) Private Nurse	0	0	279.21	4123.7	1.381	.24
(l) Optometrist	36.91	176.35	18.33	42.88	2.881	.09
(m) Dentist	141	157.77	118.85	137.63	3.835	.05
(n) Social Worker	39.39	279.19	22.25	101.95	0.234	.628
(o) Children's Aid Worker	2.01	19.08	16.3	213.98	0.002	.961
(p) Adolescence/School Counselor	6.84	61.15	0	0	4.375	.036
(q) Family Counselor	16.76	142.55	1.81	24.96	2.771	.096
(r) Mental Health Counselor	29.87	273.42	1.61	25.73	1.958	.162
(s) Homemaker/Personal Support Worker	256.35	1549.7	991.01	7655.1	3.943	.047
(t) Child/Daycare	389.22	1467.4	706.49	3476.4	1.623	.203
(u) Subsidized Daycare	38.73	425.73	52.78	396.89	0.873	.35
(v) Naturopath/Homeopath	8.1	45.57	6.48	40.11	1.016	.313
(w) Complementary Therapy	0.54	5.4	21.26	211.77	0.152	.697
(aa) Police	1.37	14.32	0	0	2.91	.088
(dd) Social and Recreation Programs	316.35	876.32	233.28	533.16	1.424	.233
(ee) Community Support Programs	8.83	108.05	14.41	129.08	0.137	.711
(ff) Special Education Services	1226.2	1448.1	995.43	1362.8	2.34	.126
(gg) Other Special Education Supports	140.37	391.31	145.7	657.27	0.312	.576
(hh) Others Social and Health providers	1264.7	5597.9	758.45	2663.1	0.512	.474

*Other Health and Social providers*	5646.6	8389.6	6203.8	11640	0.032	.857

*Community Support Services*						
(a) Groups/Peer Support	0.37	2.84	0	0	5.847	.016
(c) Transportation Services	11.43	45.59	12.2	46.45	0.22	.639
Other community Support Services	42.2	174.66	29.46	126.15	0.057	.811

*Total Community Support Services*	54	178.05	41.65	131.72	0.031	.861

*Outpatient lab tests*						
(a) Blood	20.47	75.74	16.39	44.15	0.137	.711
(b) Specimens	5.14	31.63	3.42	12.71	0.152	.697
(c) Scopes	0	0	0.61	9.74	0.689	.407
(d) X-rays	5.38	15.49	8.01	27.67	0.061	.805
(e) Scans	6.77	29.36	7.18	30.57	0.055	.814
(f) Breathing tests	0.82	5.95	0.97	5.37	0.857	.355
(g) ECG	0.79	4.43	0.62	4.27	0.421	.516
(h) EEG	2.94	14.06	1.35	8.69	1.618	.203
(i) EMG	0	0	1.2	13.54	1.381	.24
(l) Other outpatient tests	14.77	52.54	15.13	59.08	0.017	.896

*Outpatient laboratory tests*	57.09	160.66	54.88	105.6	0.258	.611

*Medications, treatments and Supplies/Aids*						
Medication	490.11	1927.5	338	963.52	0.127	.721
Treatment costs total	23.71	183.61	115.52	1714.9	0.032	.859
Supply and device cost	342.61	1329.5	1100.5	7090	2.299	.129

*Medication, treatments, supplies, device, aids*	856.43	2440.7	1554	7536.4	0.003	.956

*Direct Costs excluding Hospital stay, Day surgery*						
Direct Costs excluding Hospital	6947.8	9281	8217	14542	0.019	.891

*Hospital, Day surgery facility*						
Hospital Cost	333.13	2273.4	359.24	1950.3	0.719	.397
Day Surgery Facility Stay cost	11.43	51.26	16.54	55.19	1.505	.22
Respite	475.37	1642.4	425.57	1610	0.338	.561

*Direct Costs including Hospital stay, Day surgery*						
Total Direct Costs including Hospital/respite	7767.7	9817.2	9018.4	14992	0.175	.676

## References

[1] Varni J, Limbers C, Burwinkle T (2007). Impaired health-related quality of life in children and adolescents with chronic conditions: a comparative analysis of 10 disease clusters and 33 disease categories/severities utilizing the PedsQL 4.0 Generic Core Scales. *Health and Quality of Life Outcomes*.

[2] Majnemer A, Shevell M, Rosenbaum P, Law M, Poulin C (2007). Determinants of life quality in school-age children with cerebral palsy. *The Journal of Pediatrics*.

[3] Aran A, Shalev RS, Biran G, Gross-Tsur V (2007). Parenting style impacts on quality of life in children with cerebral palsy. *Journal of Pediatrics*.

[4] Arnaud C, White-Koning M, IShoy Michelsen S (2008). Parent-reported quality of life of children with cerebral palsy in Europe. *Pediatrics*.

[5] Klassen AF, Miller A, Fine S (2004). Health-related quality of life in children and adolescents who have a diagnosis of attention-deficit/hyperactivity disorder. *Pediatrics*.

[6] Seid M, Varni JW, Segall D, Kurtin PS (2004). Health-related quality of life as a predictor of pediatric healthcare costs: a two-year prospective cohort analysis. *Health and Quality of Life Outcomes*.

[7] Varni JW, Seid M, Rode CA (1999). The PedsQL: measurement model for the pediatric quality of life inventory. *Medical Care*.

[8] Chan KS, Mangione-Smith R, Burwinkle TM, Rosen M, Varni J (2005). The PedsQL: reliability and validity of the short-form generic core scales and asthma module. *Medical Care*.

[9] Varni JW, Burwinkle TM, Seid M, Skarr D (2003). The PedsQL as a pediatric population health measre: feasibility, reliability and validity. *Ambulatory Pediatrics*.

[10] Statistics Canada National longitudinal survey of children and youth. http://www.statcan.gc.ca.

[11] National longitudinal survey of children and youth, cycle 7—user’s guide. http://www.statcan.gc.ca/imdb-bmdi/document/4450_D4_T9_V7-eng.pdf.

[12] Kessler R, McGonagle K, Zhao S (1994). Lifetime and 12-month prevalence of DSM-III-R psychiatric disorders in the United States: results from the from the national comorbidity survey. *Archives of General Psychiatry*.

[13] Kessler RC, Andrews G, Colpe LJ (2002). Short screening scales to monitor population prevalences and trends in non-specific psychological distress. *Psychological Medicine*.

[14] Statistics Canada http://www.statcan.gc.ca.

[15] Strayhorn JM, Weidman CS (1988). A parenting practices scale and its relation to parent and child mental health. *Journal of the American Academy of Child and Adolescent Psychiatry*.

[16] Byles J, Byrne C, Boyle MH, Offord DR (1988). Ontario Child Health Study: reliability and validity of the general functioning subscale of the McMaster Family Assessment Device. *Family Process*.

[17] Cutrona CE, Russell DW (1987). The provisions of social relationships and adaptation to stress. *Advances in Personal Relationships*.

[18] Stein RE, Riessman CK (1980). The development of an impact-on-family scale. *Medical Care*.

[19] Stein RE, Jessop DJ (2003). The impact on family scale revisited: further psychometric data. *Journal of Developmental and Behavioral Pediatrics*.

[20] Williams A, Piamjariyakul U, Williams P, Bruggeman S, Cabanela R (2006). Validity of the revised impact on family (IOF) scale. *Journal of Pediatrics*.

[21] Browne G, Arpin K, Corey P, Fitch M, Gafni A (1990). Individual correlates of health service utilization and the cost of poor adjustment to chronic illness. *Medical Care*.

[22] Browne G, Gafni A, Roberts J Approach to the measurement of resource use and costs.

[23] Browne G, Roberts J, Gafni A (1999). Economic evaluations of community-based care: lessons from twelve studies in Ontario. *Journal of Evaluation in Clinical Practice*.

[24] Browne G, Roberts J, Weir R, Gafni A, Watt S, Byrne C (1994). The cost of poor adjustment to chronic illness: lessons from three studies. *Health & Social Care*.

[25] Spitzer WO, Roberts R, Delmore T (1976). Nurse practitioners in primary care. V: development of the utilization and financial index to measure effects of their deployment. *Canadian Medical Association Journal*.

[26] Petrou S, Murray L, Cooper P, Davidson L (2002). The accuracy of self-reported healthcare resource utilization in health economic studies. *International Journal of Technology Assessment in Health Care*.

[27] Guerriere D, Wong A, Croxford R, Leong V, McKeever P, Coyte P (2008). Costs and determinants of privately financed home-based health care in Ontario, Canada. *Health and Social Care in the Community*.

[28] Guerriere DN, Ungar WJ, Corey M (2006). Evaluation of the ambulatory and home care record: agreement between self-reports and administrative data. *International Journal of Technology Assessment in Health Care*.

[29] Cohen J (1988). *Statistical Power Analysis for the Behavioral Sciences*.

[30] Varni JW, Burwinkle TM, Berrin SJ (2006). The PedsQL in pediatric cerebral palsy: reliability, validity, and sensitivity of the Generic Core Scales and Cerebral Palsy Module. *Developmental Medicine and Child Neurology*.

